# Reactive Powder Concrete Microstructure and Particle Packing

**DOI:** 10.3390/ma15062220

**Published:** 2022-03-17

**Authors:** Evgeny Georgievich Velichko, Nikolai Ivanovich Vatin

**Affiliations:** 1 Department of Building Materials, Moscow State University of Civil Engineering, 129337 Moscow, Russia; strictly@mail.ru; 2 Peter the Great Saint Petersburg Polytechnic University, 195251 Saint Petersburg, Russia

**Keywords:** dispersed composition, mineral modifiers, strength, concrete, cement

## Abstract

The subject of this study is the dispersed composition of multicomponent cement systems. This study aims to reduce interparticle voids, increasing the strength and concentration of the solid phase. The investigated concrete mixture contained two fine aggregate fractions, granite-gabbro crushed stone of 5–10 mm fraction, Portland cement of CEM I 42.5N class, finely dispersed granular blast furnace slag, microsilica, highly dispersed cement fraction, superplasticizer Glenium 430, and high-valence hardening accelerator. A laser analyzer determined the shape and size of dispersed particles of the components. The structure of the cement stone was studied by scanning microscopy, thermographic, and X-ray phase analysis methods. The strength of concrete with an optimized dispersed composition at the age of 2 days was 52, 63, and 74 MPa, while that at the age of 28 days was 128, 137, and 163 MPa. For this concrete, the consumption of multicomponent cement was 650, 700, and 750 kg/m^3^, respectively. The high efficiency of the application of bimodal clinker component and granulated blast-furnace slag is shown. It is established that the optimal content of nanoscale additives, including microsilica, should be insignificant and determined experimentally.

## 1. Introduction

The disadvantages of high-performance concrete (HPC) and ultrahigh-performance concrete (UHPC) are the binder’s high absolute and specific consumption per strength unit. HPC and UHPC have achieved the maximum compressive strength in their existing components’ structure. However, with a further increase in the load, the coarse aggregate becomes the weakest part of the concrete. The only solution is to remove the coarse aggregate to increase the compressive strength of concrete even further. The next step in increasing the compressive strength of the concrete is to remove the coarse aggregate. Coarse aggregate is absent in reactive powder concrete [1]. Comparing reactive powder concrete’s physical, mechanical, and durability properties with those of HPC/UHPC shows that reactive powder concrete possesses better strength (both compressive and flexural) and lower permeability than HPC.

The use of steel and polypropylene fibers improves the strength characteristics of all types of concrete and dispersed reinforcement. Therefore, the hack-characteristics of concrete can be further analyzed without taking into account the reinforcement [2,3,4].

A promising direction for reactive powder concrete production is the use of self-compacting concrete (SCC) [5,6,7]. The self-compacting concrete mix should be characterized by high viscosity and fluidity; there should be no water separation and delamination. The use of superplasticizers of the polycarboxylate type, finely dispersed mineral materials, viscosity modifiers, set retarders, and hardening accelerators provides such characteristics.

For low yield stress, the self-compacting concrete mixture includes an increase of up to 0.5 quota of sand in a concrete mixture, a decrease of up to 3–10 mm in the maximum size of a coarse aggregate, and a low water content. High fluidity and self-compacting of the concrete mixture are achieved by reducing contact interactions between aggregate grains, as well as a high content of cement paste [7,8,9,10].

Concretes with a high cement content have an increased heat release [11]. For this reason, the fixation of the newly formed particles of hydrate phases during structure formation occurs due to molecular selection, mainly in the position of the weak flocculation [12,13] in the framework of the Derjaguin–Landau–Verwey–Overbeek (DLVO) theory [14]. This type of fixation determines an increase in the cement paste’s defectiveness, looseness, and microporosity, and a decrease in the building properties of concrete [15].

It is possible to obtain concrete mixtures with a high cement paste content at lower consumption of clinker cement with its partial replacement by highly dispersed mineral additives (finely ground granulated blast furnace slag, fly ash, microsilica, etc.) [16]. However, it is necessary to consider the possible dependence of the parameters (dispersity, content) on the shape and size of the particles of clinker cement using mineral additives. The authors of [17,18,19] discussed the increase in the activity of mineral additives by increasing the specific surface area developed thermodynamic and mathematical models for optimizing the composition of composite mixtures. The main problem of concrete modified with mineral additives is to optimize their dispersed composition, taking into account the spatial and geometric parameters (dispersion of particles, geometry, volume, energy state, the average size of interparticle voids, their fractality, isometrics, etc.) of the clinker component and its pozzolanic activity.

Considering the spatial and geometric parameters of clinker and mineral additives makes it possible to increase the strength of concrete and other structural properties of concrete. When replacing part of the cement with mineral additives, it is necessary to take into account the ratio of sizes (dispersion) of clinker particles and particles of mineral additives. The key factor is using different dispersed mineral additives in one concrete mix. Pozzolanic activity and the energy state of mineral additives also significantly affect the solid phase concentration per unit volume of the concrete mixture [20].

Mineral additive particles of each subsequent more finely dispersed fraction should be placed in between larger particles or grains, providing the maximum possible filling of voids. Small mineral particles with a tessellated distribution on the exterior of clinker particles, large mineral additives, and Coulomb reciprocal action between particles lead to their spontaneous orientation in the space between larger particles. They are hooked with high binding energy in the interparticle voids of a multicomponent system at their hierarchical dispersed granulometric levels of the mix structure. The binding energy increases with decreasing particle size; hence, dispersity can be used to increase the cement paste density. The self-organization of the concrete mixture’s structure decreases the disarrangement of the system’s component, increasing the particles’ potential energy and viscosity. All of this ensures high homogeneity and non-delamination of self-compacting concrete. The volume of the remaining voids in such a system will be minimal [20,21].

Thus, obtaining self-compacting concrete of high strength classes can be ensured only by synthesizing a homogeneous optimized discrete–continuous dispersed-particle size distribution of particles and grains of the solid phase in each hierarchical structural level of the material. The scanning electron microscopy, thermographic, and X-ray phase analysis methods were used to study the structure of the cement paste [21]. It was shown that it is commendable to use clinker particles of several levels of dispersion. The homogeneous optimized approach was proposed to design the balanced content of the concrete mixture with a marked up solid-phase concentration. The approach is based on the rational ratio of highly dispersive mineral and superplasticizers that optimize concrete mixture composition and structure.

The sufficient amount of empirical data on reactive powder concrete microstructure gives possibilities to suggest four commonly used mixture design methods for UHPC [22]. These methods include close packing methods based on dry and wet packing densities [23,24,25], method, based on the relationship between rheological properties of paste and raw materials [26,27], statistical analysis methods [28], and artificial neural network (ANN) models [29,30]. The packing of solid particles is used for mixture design in ceramic processing [31,32,33], asphalt concrete [34], and construction concrete [35,36,37].

It follows from the above review that there are a sufficient amount of empirical data on the microstructure of reactive powder concrete. However, these data did not lead to creating a unified theory and design model of the concrete mix. This article aims to suggest a simple model to optimize the ratio of finely dispersed clinker particles and mineral additives. The objectives are the experimental study of multicomponent cement paste and optimizing the dispersed composition of concrete modified with mineral additives to reduce interparticle voids with a significant increase in the solid phase concentration per unit of volume and strength.

## 2. Materials and Methods

The properties and structure of concrete were studied using two fractions of fine aggregate 0.315 and 0.16 mm in size, respectively, in the amounts of 80% and 20%, granite-gabbro crushed stone of 5–10 mm size, Portland cement of the CEM I class 42.5 N with a specific surface area of 253 m^2^/kg, finely dispersed granular blast furnace slag with a specific surface area of 423 m^2^/kg, microsilica, and fine fraction of cement. Cement consumption, including multicomponent, depending on the research task, varied in the range of 450–750 kg/m^3^ of concrete. As a superplasticizer, Glenium 430 (OOO BASF-Stroitelnye Sistemy, Moscow, Russian) was used in the amount of 0.45–0.56% of the cement mass, and a high-valence hardening accelerator AC was used in the amount of 0.07% following the Schulze–Hardy rule [13,38].

The structure of the cement stone was studied with an ARL X’TRA X-ray Diffractometer, Termo Fceser Scientifie Ins. Waltham, Massachusetts, USA. Differential thermal analysis was achieved with a DTG–60 thermal analyzer, Shimadzu Co, Kyoto, Japan. Structure microanalysis was completed with a Quanta 200 FEG scanning electron microscope, Thermo Fisher Scientific, Waltham, MA, USA.

Multiple research methods were used. The shape and size of dispersed particles of the components were determined by the sedimentation method according to Russian State Standard GOST 8269.0–97 “Mountainous rock road-metal and gravel, industrial waste products for construction works methods of physical and mechanical tests” [39] and by a laser analyzer according to Russian State Standard GOST P 57923–2017 “Ceramic composites. Determination of particle size distribution of ceramic powders by laser diffraction method” [40]. The mobility of the concrete mixture was determined according to Russian State Standard GOST 10181–2014 “Concrete mixtures. Methods of testing” [41]. The compressive strength of concrete was determined according to Russian State Standard GOST 10180–2012 “Concretes. Methods for strength determination using reference specimens” [42].

## 3. Results

The study of the structure using scanning microscopy with microanalysis showed that finely ground granulated blast furnace slag with an optimal amount and fineness has a uniform distribution (coefficient of variation less than 0.5%) of its particles in the matrix of a multicomponent cement paste.

**Figure 1:**
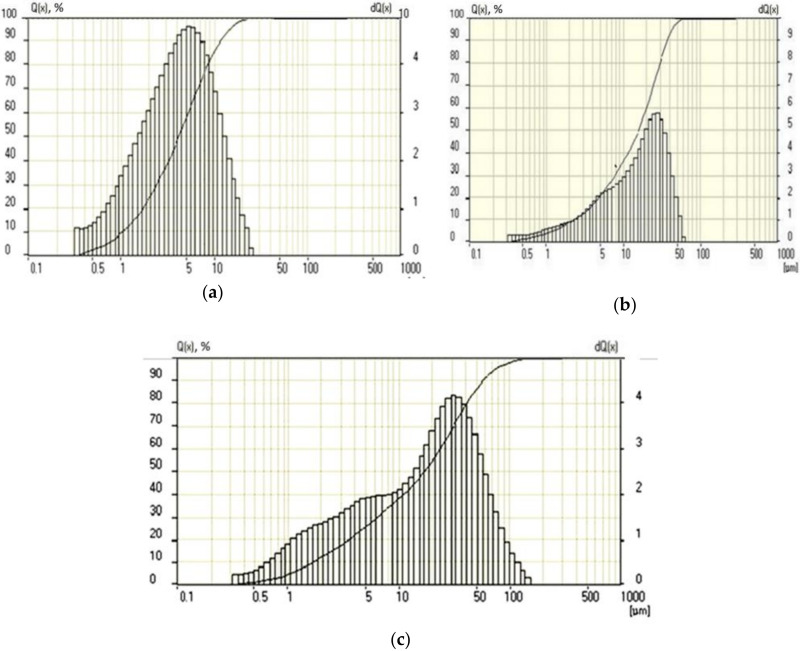
Particle size distribution function *Q(x)* and a probability density *dQ(x)*: (**a**) finely dispersed cement (1000 m^2^/kg); (**b**) conventional Portland cement; (**c**) finely ground granulated blast furnace slag.

The finely dispersed clinker component for the multicomponent cement was used with 950–1000 m^2^/kg dispersion. Particles with a size of 1–5 microns and less constituted up to 50%, and particles up to 10 microns constituted up to 76%. This fine fraction content and the used clinker component were 2.5 times higher than their content in industrial Portland cement (Figure 1). The average particle sizes of conventional Portland cement, fine slag, and fine cement were 35, 20, and 3.5 μm, respectively.

The functional necessity of using fine cement is as follows: firstly, this ensures a uniform reaction between portlandite and silicon dioxide in all microvolumes of the cement paste. Such homogeneity of reactions is a more uniform distribution of new hydrosilicate phase formations in each cement paste microvolume, with significantly lower defectiveness and high concrete strength. Secondly, the second concentration level fills the solid-phase concentration per unit volume. Thirdly, this decreases the degree of hydration of particles of the coarse fraction of the clinker component, large durable relics, which significantly increases the strength of the cement paste and the durability of concrete. Fourthly, this is the early activation of the hydration of slag minerals.

**Figure 2:**
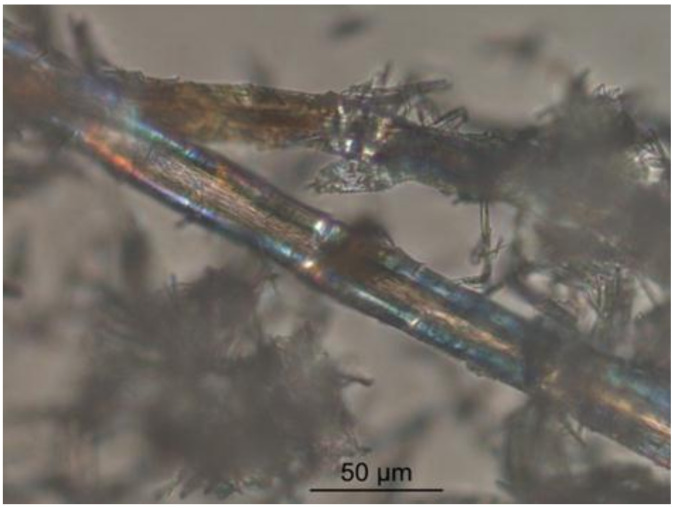
Threadlike nanocrystals of ettringite.

**Figure 3:**
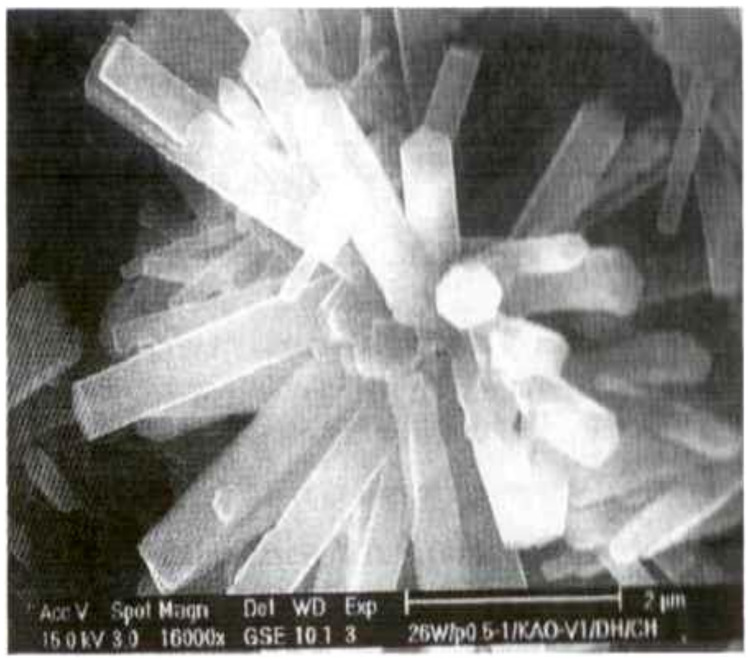
Crystals of ettringite with hexagonal prismatic morphology.

**Figure 4:**
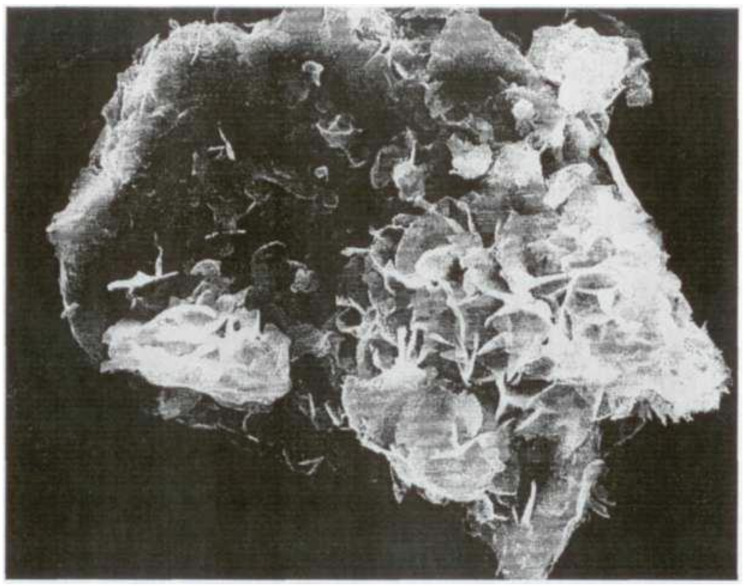
Tricalcium aluminate particles covered with needle-shaped crystals of calcium hydroaluminates.

The authors present a hypothesis to explain the phenomena under consideration. Highly dispersed clinker particles are almost completely hydrated at the initial period of cement’s hydration. These particles are mainly of the second hierarchical level of the multisize cement particles. As a result of molecular selection, the hydration products of these particles are scattered into the entire volume of the hydrated phases corresponding to them in the composition and structure of new formations of neighboring larger particles. At the same time, the hydration process of mineral additives is activated. The layer of hydrosilicate gel congeals on the outside of the unhydrated surface of the cement particle, captivating crystals of ettringite, and the cement paste’s microstructure grow to form conglomeration containing clinker particles as crystallization centers. Calcium hydrosulfoaluminates are impurity inclusions in the microstructure of the potassium silicate hydrate phase. They contribute to an increased hydrate phase’s density and strength due to their nanometer size and the possibility of the formation of percolations of chains/clusters and fibriform (Figure 2), needle-shaped, or hexagonal-prismatic inclusions in the structure in the presence of superplasticizer in the concrete mixture (Figure 3 and Figure 4). The length of ettringite crystals is 1000–3000 nm, and their cross-sectional size ranges from 40 to 120 nm. The crystals can reach lengths of up to 8 µm with a cross-section of up to 220 nm.

The energetic interaction of the formed chains/clusters of calcium hydrosulfoaluminates with new hydrosilicate phases contributes to their fixation in the densest packing, providing a significant contribution to the synthesis of the strength of the cement stone (first proposed by the authors of this work). Their simultaneous inclusion as an impurity phase reduces the strength of new hydrosilicate phases of cement stone. Thus, the contribution of calcium hydrosulfoaluminates to the strength of the cement stone structure is dual. Therefore, it is advisable to use cement with a low tricalcium aluminate content for high-strength concretes.

Calcium hydroxide is released from the hydration of calcium silicate minerals of clinker. Calcium hydroxide reacts with silica fume as an amorphous polymorph of silicon dioxide and other silicious components, mainly in an amorphous form, composing strong low-basicity secondary calcium hydrosilicates. In this case, the hydration processes of the clinker minerals C3S and C2S are accelerated. Later, the product of the pozzolanic reaction of the lower-kinetic-energy particles of the hydrate phases has significantly less deficiency, a higher degree of structure ordering, and high density and strength due to close coagulation of these particles.

With a low degree of disorder, their compressive strength can reach 1000 MPa. This product’s chemically bound water content is 1.5–2 times lower than in primary neoplasms of hydrosilicate phases [19]. Later, the products of hydration of the calcium silicate phases of clinker, slag, and pozzolanic reaction coalesce into a durable, uniform, high-dispersity structure of a conglomerate or composite type. The pores in such a dense structure are mostly helium-sized. C–S–H with different stoichiometry, residuals of clinker, slag, and hard particles of mineral additives ensure the strength of this structure.

**Figure 5:**
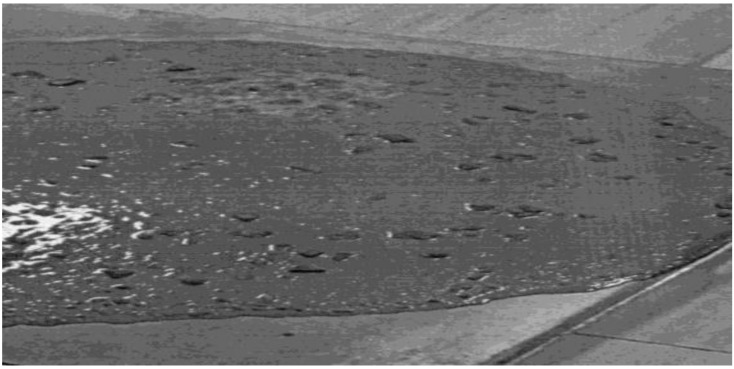
Self-compacting concrete mix with 88 cm cone flow.

Thus, for a significant increase in the strength of reactive powder concrete, a multilevel particle packing of a multicomponent concrete mixture with a regularity of the particle size distribution is required. Complete hydration of the clinker particles’ minerals is not required to ensure the high strength of such a structure. The SCC mixture with a cone flow diameter of 88 cm, prepared following the previously stated provisions, has low yield stress, high deformability, the absence of water gain, gravity segregation, and separation stability. There are uniformly distributed grains of coarse aggregate on the surface of the concrete mixture, confirming the properties mentioned above (Figure 5).

Experimental studies of the authors showed the optimal amount of highly dispersed cement at 6% and microsilica at 3%. It was also found that the optimal content of the superplasticizer GleniumACE 430 is 0.45–0.56% of the cement mass, and that of the high-valence hardening accelerator AC is 0.07%. The optimum content of the hardening accelerator AC was determined, taking into account the Schulze–Hardy rule [22]. The use of a hardening accelerator provided a synergistic effect of using Glenium 430 and made it possible to additionally reduce the water content of the concrete mixture up to 20%. The introduction of a hardening accelerator into a concrete mixer must be carried out after the plasticizer for 8–10 s, before the end of the preparation of the concrete mixture, which ensures its adsorption on the grafted side chains of the polycarboxylate superplasticizer and a significant increase in its plasticizing reduction effect due to the increase in the total repulsive forces in concrete modified with mineral additives.

Studies have also shown that the decrease in the optimized and self-organized interparticle void content at four dispersed levels of concrete modified with mineral additives due to the optimal spatial, geometric, and quantitative parameters of mineral additives, as well as the almost mosaic energy state of the surface of the original powder components, is 12–14% or more, with a more than twofold increase in the strength of concrete. The contribution to reducing the interparticle void content of real concrete modified with mineral additives only due to finely dispersed slag, depending on its content, is more than 9%, which is in good agreement with the theoretical value (8.09%). In particular, the compressive strength of concrete at the age of 2 days after hardening under normal conditions was 52, 63, and 74 MPa, while that at 28 days was 128, 137, and 163 MPa, with 650, 700, and 750 kg/m^3^ consumption of multicomponent cement, respectively.

**Figure 6:**
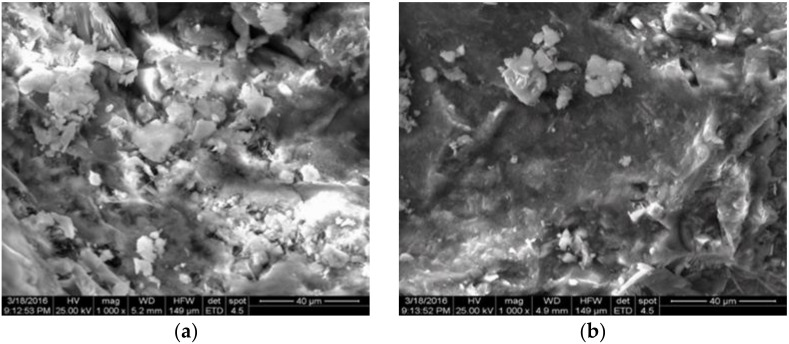

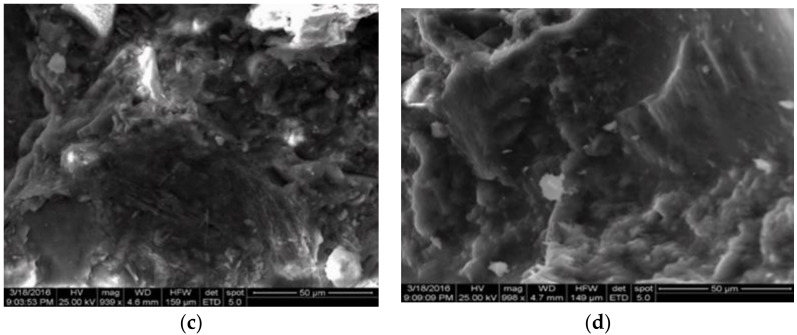
The structure of the modified multicomponent cement stone after hardening under normal conditions at the age of 28 days: (**a**) control composition; (**b**) with fine slag; (**c**) with fine slag and floured cement; (**d**) with fine slag, floured cement, and microsilica.

Studies were carried out using scanning electron microscopy to assess the quality of the concrete structure (Figure 6).

**Figure 7:**
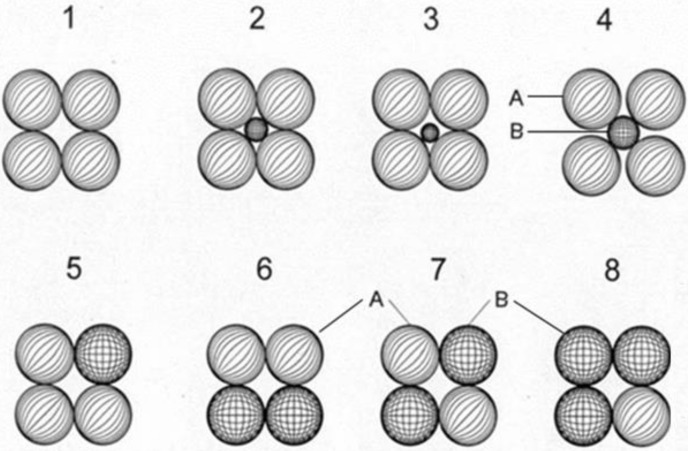
Assumed particle distribution of the clinker component and mineral additives. A is a clinker component; B is a mineral additive; 1 clinker components without mineral additives; 3 optimal dispersion of mineral additives; 2 less than optimal dispersion of mineral additives; 4 less dispersion of large aggregates of mineral additives that of clinker components; 5–8 equal dispersion of particles of clinker component and mineral additives.

The use of mineral additives with parameters (dispersion, content), determined according to the results of this and previously published research [21] significantly improves the quality of concrete structure (Figure 7, Images 3 and 4) to a nano-modified level. Thermographic and X-ray phase studies of its samples of the structure of concrete with mineral additives at the age of 28 days after hardening under normal conditions and steam curing were also carried out (Table 1).

The structure of concrete with mineral chemical modifiers, which have optimal parameters, is distinguished by a significantly lower content of portlandite, and the degree of hydration of Portland cement is 83–85%, exceeding the same indicator for the control composition by 20–27%, confirming its higher strength.

## 4. Discussion

The close parking method of the UHPC mixture design based on packing density refers to the proportion of solid volume to the cement and additive system’s total volume [22,23,24]. The authors propose a physical model of concrete microstructure and particle packing, consisting of spherical particles, to explain the results obtained. Figure 7 shows the assumed particle distribution of the clinker component and mineral additives. The author in [15] previously published a similar picture as a hypothesis without experimental confirmation.

The unit cell of the particles of the clinker component is presented in the form of a simple pseudo-cubic packing of particles of the same diameter (Figure 7, Image 1). The clinker components’ matrix is assumed to be a simple cubic packing with voidness of 48%, consisting of particles of the same diameter. This assumption is substantiated by the fact that the voidness of real cement powders is 55–58%. Furthermore, the results of sedimentation and laser analyses of the dispersed composition of various types of cement and mineral additives showed that the interval of 12–15 µm accounts for up to 85% of their distribution.

It can be assumed that there are three cases of dispersion of mineral additives to increase the density of concrete modified with mineral additives:

(1) The dispersion of mineral additives is optimum in the sense that the mineral additive particles fill the voids between the clinker particles (Figure 7, Image 2). In this case, the increase in the cement paste density will be by 8.09% more than without mineral additives (Figure 7, Image 1).
(2) The dispersion of clinker particles and mineral additive particles is the same. The density of the original cement paste does not change if the mineral additive is added (Figure 7, Images 5–8).
(3) The dispersion of particles of mineral additives significantly exceeds the dispersion of clinker particles (Figure 7, Image 3). An example of such a finely dispersed mineral additive is microsilica with a specific surface area of 18,000–21,000 m^2^/kg. In this case, the increase in the cement paste density could be more than without mineral additives. However, increased mineral additive content could also lead to the formation of large stable aggregates of small microsilica particles with a high binding energy between particles. These large aggregates of mineral additives can lead to the decompaction of clinker particles (Figure 7, Image 4), an increase in its interparticle voidness, and consequently a negative impact on the properties of concrete. In cement systems of a conglomerate or composite type of structure, especially in the presence of variously dispersed mineral additives, there is a possibility that two or more dispersed particles, i.e., mineral additives, may combine and form a separate aggregate [2,19]. In such microvolumes of cement systems, the pozzolanic reaction practically does not occur. These microvolumes are pseudopores of 5–7 µm (when three particles are combined) and 0.5–1.5 µm (when two particles are combined). With a large number and insignificantly small size of particles and the formation of large aggregates, the particles can be distributed in the interparticle voids of the elementary cells of the matrix component, loosening them (Figure 7, Image 2). The indicated pseudopores are structural defects and will significantly decrease strength, frost resistance, and deformation characteristics of concrete modified with mineral additives.

The positive and negative influence of mineral additives on the concrete’s structure formation and properties requires determining its optimal content in the concrete composition. The rational content of mineral additives for the first case could be 18–25%. The second case with equal dispersion of the clinker component and mineral additives requires 25%, 50%, and 75% content of mineral additives. For the third case, the content of mineral additives is small and is determined experimentally.

Pozzolanic activity of mineral additives affects the degree of hydration of clinker minerals, the nature of the products of hydrate phases, the formation of the properties of the contact zone between the particles of mineral additives and neoplasms, and the integral strength of concrete modified with mineral additives. If inactive mineral additives are used, then the amount of replaced cement decreases, and the content of mineral additives increases. In the case of equivalent replacement of cement with highly active mineral additives and obtaining the strength of concrete modified with mineral additives on their basis higher than the strength of the control composition, the content of cement and mineral additives should decrease in a given ratio in proportion to the increase in strength, thus maintaining the uniformity of the distribution of the particles that form the matrix.

If finely dispersed slag is used as the mineral additive, which is capable of independent hydraulic hardening, then its particles should also be distributed mainly in the interparticle voids of the clinker component. The rational content of slag in the composition of the cement mixture is 35–70% [11]. This content is greater than the volume of interparticle voids of the clinker component. Therefore, it is advisable to use a bimodal particle size distribution or slag or clinker component to obtain a high-density concrete modified with mineral additives of dispersed composition.

An example of the bimodal dispersion of clinker and mineral practices (Figure 7, Image 2) could be the use of 40% (by volume) slag and a clinker component of equal dispersion and 20% slag or clinker component with a higher dispersion. In this case, the maximum filling of concrete modified with mineral additives with finely dispersed granular blast furnace slag of bimodal particle size can be up to 60% and even up to 80%. Only single-mode granular blast furnace slag and a more highly dispersed clinker component with an optimal specific surface area could be used in the latter case.

It is possible to use highly dispersed nanosized mineral additives (carbon fibers, fullerenes, nanosilica, etc.) to obtain high-strength self-compacting concrete. Their content should be insignificant. The increased content of nanosized mineral additives can decrease the positive effect of their use due to the aggregation of nanosized particles.

The above-proposed physical model of concrete microstructure and particle packing is confirmed by the presented and previous [15,16] experimental results. Thus, the following guidelines are proposed for obtaining self-compacting concrete modified with mineral additives: coarse aggregate should be present in fractions of 5–10 mm. The content of coarse aggregate in the concrete mixture should not exceed a fraction (0.5) of sand to ensure a low level of the ultimate shear stress of the concrete mixture [43]. The fine aggregate should have two or three fractions, for example, a coarse fraction of 0.3 mm in an amount of 80% and a fine fraction of 0.12 mm in an amount of 20%. Such a fractional composition of the fine aggregate provides a decrease in its intergranular voidness and a decrease in air entrainment into the concrete mixture.

The total volume of a multicomponent binder could be determined using the absolute volume method. The dispersion of a multicomponent binder has three levels. For example, the first level could be Portland cement with 220–250 m^2^/kg or less dispersion. This coarse cement fraction ensures the long-term preservation of strong relics of clinker particles with a high modulus of elasticity in concrete. The finely dispersed granulated blast furnace slag should be in an amount of 20–25% of the cement mass with the dispersion of 380–450 m^2^/kg to fill the first level of interparticle voids of Portland cement.

For the second level, finely dispersed Portland cement could be used in an amount of 4–12% with the dispersion of 900–1100 m^2^/kg. This dispersion ensures a uniform course of the pozzolanic reaction in all micro volumes containing mineral modifiers particles and prevents aggregates of particles of mineral modifiers.

For the third level, microsilica could be used in the amount of 2–6% with the dispersion of 18,000–21,000 m^2^/kg. Microsilica percolates between larger particles. The fixation of particles of neoplasms of hydration phases occurs due to molecular selection by strong flocculation [12,13] at a distance of 10^−9^ m between the particles. The strong flocculation significantly increases the density and strength of concrete modified with mineral additives. In addition, due to the pozzolanic reaction, strong dendrite-like secondary low-basicity hydrosilicate phases are formed. This is because highly dispersed amorphous silicon dioxide, interacting with calcium hydroxide, forms fibrous and tubular nanosized calcium hydrosilicates almost during the preparation of the concrete mixture. These hydrosilicates of calcium create agglomerations that facilitate the clustering of particles of neoplasms of hydrate phases in high-density packing. The amount of microsilica, in this case, is small and is determined experimentally. Using a multicomponent highly dispersed binder requires the mandatory use of polycarboxylate-type superplasticizers and, preferably, high-valence hardening accelerators.

In addition, with a high cement content, the total alkali content in various components of the concrete composition could exceed the critical value of 3 kg/m^3^. High alkali content requires aggregates containing reactive silica to provide alkaline corrosion of the aggregates. Consequently, using limestone and dolomite flour in concrete modified with mineral additives is less effective than mineral additives containing reactive silica.

## 5. Conclusions

This study shows that the determining factor in obtaining a homogeneous, highly filled, dense structure of a cement stone with low defectiveness is the use of heterogeneous mineral additives only with a specific surface area and content that are functionally related to the spatial geometric and energy state of the parameters of the particles that form the interparticle voids of the matrix component. With optimal values of geometric and quantitative parameters using mineral additives, the initial interparticle void content of concrete modified with mineral additives is reduced by 12–14% or more, and the strength of concrete is more than doubled. The volume of interparticle voids decreases by 9% or more due to the use of finely dispersed slag.

It is advisable to use particles of the clinker phase of several geometrical sizes, providing an increase in the concentration of the solid phase in a unit volume, a homogeneous course of pozzolanic, and hydration reactions of clinker minerals in all microvolumes of concrete modified with mineral additives. In addition, there is also a significant increase in the density of the cement stone, and the presence of large, strong relics of the particles of the clinker component at a later date, significantly contributing to the integral strength and durability of concrete.

The high efficiency of the complex application of polycarboxylate-type superplasticizers and high-valence hardening accelerator AC has also been established. The optimum content of the hardening accelerator AC is 0.07% of the cement mass as determined taking into account the Schulze–Hardi rule. Using a high-valence hardening accelerator provides a synergistic effect of using the superplasticizer Glenium 430. This makes it possible to additionally reduce water-containing concrete mixtures up to 20%. The introduction of the hardening accelerator into the concrete mixer must be carried out after the plasticizer, 8–10 s before the end of the preparation of the concrete mixture.

## Figures and Tables

**Table 1:** Results of thermographic and radiographic analyses.

No.	Type of Mineral Additive and Its Content, %	Weight Loss, %, at Temperature, °C			Ca(OH)_2_, %	Degree of Hydration, %
		136–147	519–531	822–832		
At the age of 28 days after hardening under normal conditions						
1	Control composition	18.7	23.1	29.2	21.9	68
2	Fine slag 20%	13.6	22.1	24.6	17.2	81
3	Fine slag 20% Microsilica 3% Fine fraction of cement 6%	13.8	14.2	20.3	15.1	85
After steam curing at the age of 28 days						
4	Control composition	15.1	16.9	24.7	21.1	63
5	Fine slag 20%	12.2	17.5	20.4	18.2	76
6	Fine slag 20% Microsilica 3% Fine fraction of cement 6%	10.3	11.6	18.1	10.9	83

## Data Availability

The data presented in this study are available on request from the corresponding author.
